# Boosting BCG with recombinant influenza A virus tuberculosis vaccines increases pulmonary T cell responses but not protection against *Mycobacterium tuberculosis* infection

**DOI:** 10.1371/journal.pone.0259829

**Published:** 2021-11-18

**Authors:** Heni Muflihah, Manuela Flórido, Leon C. W. Lin, Yingju Xia, James A. Triccas, John Stambas, Warwick J. Britton

**Affiliations:** 1 Centenary Institute, The University of Sydney, Sydney, New South Wales, Australia; 2 School of Medicine, Deakin University, Geelong, Victoria, Australia; 3 School of Medical Sciences, Faculty of Medicine and Health, The University of Sydney, Sydney, New South Wales, Australia; 4 Central Clinical School, Faculty of Medicine and Health, The University of Sydney, Sydney, New South Wales, Australia; 5 Department of Clinical Immunology, Royal Prince Alfred Hospital, Camperdown, New South Wales, Australia; Karolinska Institutet, SWEDEN

## Abstract

The current *Mycobacterium bovis* BCG vaccine provides inconsistent protection against pulmonary infection with *Mycobacterium tuberculosis*. Immunity induced by subcutaneous immunization with BCG wanes and does not promote early recruitment of T cell to the lungs after *M*. *tuberculosis* infection. Delivery of Tuberculosis (TB) vaccines to the lungs may increase and prolong immunity at the primary site of *M*. *tuberculosis* infection. Pulmonary immunization with recombinant influenza A viruses (rIAVs) expressing an immune-dominant *M*. *tuberculosis* CD4^+^ T cell epitope (PR8-p25 and X31-p25) stimulates protective immunity against lung TB infection. Here, we investigated the potential use of rIAVs to improve the efficacy of BCG using simultaneous immunization (SIM) and prime-boost strategies. SIM with parenteral BCG and intranasal PR8-p25 resulted in equivalent protection to BCG alone against early, acute and chronic *M*. *tuberculosis* infection. Boosting BCG with rIAVs increased the frequency of IFN-γ-secreting specific T cells (p<0.001) and polyfunctional CD4^+^ T cells (p<0.05) in the lungs compared to the BCG alone, however, this did not result in a significant increase in protection *against M*. *tuberculosis* compared to BCG alone. Therefore, sequential pulmonary immunization with these rIAVs after BCG increased *M*. *tuberculosis*-specific memory T cell responses in the lung, but not protection against *M*. *tuberculosis* infection.

## Introduction

Tuberculosis (TB) is the commonest cause of death from an infectious disease globally and was responsible for one billion deaths over the last two centuries [1] and 1.4 million death in 2019 [2]. The current TB vaccine *Mycobacterium bovis* Bacille Calmette-Guérin (BCG) provides reliable protection against severe TB disease in children, but only variable protection against pulmonary TB in adults [3, 4]. In a population at high risk for TB, neonatal BCG vaccination provided protection for only ten years [5, 6], and the waning of BCG efficacy contributes to the increase in TB cases observed in adolescents and young adults [7, 8]. The variability and limited durability of the protective immunity generated by BCG also limit its impact on the transmission of *M*. *tuberculosis*. The failure of BCG to provide long-lived protection against *M*. *tuberculosis* infection is related to the waning of the BCG-induced immune response. For example, in mice the level of cytokine secreting antigen-specific CD4^+^ T induced by BCG significantly declines by 16-weeks post-immunization [9]. The long-term efficacy of BCG may be improved by boosting BCG with subunit vaccines to sustain the level of protective immunity [8], and a variety of viral and protein/adjuvant vaccines are being evaluated as boosting vaccines in preclinical models [10, 11].

One approach for enhancing the efficacy of BCG priming is to deliver the boosting vaccine to the lungs, the primary site of encounter with *M*. *tuberculosis*. This requires efficient delivery systems. Viral-vectored TB vaccines present one approach for delivery to the lungs [12], and recently protein/adjuvant vaccines with adjuvants approved for human use have also been safely delivered as pulmonary vaccines [13]. Both modified vaccinia virus expressing the *M*. *tuberculosis* Ag85A (MVA85A) and replication deficient adenovirus expressing Ag85A boosted the protective efficacy of BCG in mice when delivered by the intranasal (i.n.), but not the parenteral route [10, 14]. The route of delivery was essential for this effect [14], and subsequently, boosting with intramuscular delivery of the MVA85A vaccine failed to increase the protective efficacy of BCG in a Phase IIB clinical trial in infants [15]. Further, boosting BCG-vaccinated infants with intramuscular adenovirus AERAS-402 vaccine failed to generate a strong polyfunctional CD4^+^ and CD8^+^ T cell responses [16]. This contrasts with results from preclinical studies where pulmonary delivery of TB vaccines to boost BCG stimulated the strongest antigen-specific T cell responses in the lungs compared to immunization by other routes [17, 18]. These results highlight the need for novel TB vaccines suitable for pulmonary delivery in humans.

Simultaneous immunization (SIM) with vaccines given by the pulmonary and parenteral routes may activate local mucosal immune responses that inhibit early *M*. *tuberculosis* infection and systemic immunity provides sustained protection. For example, SIM with subcutaneous BCG and intranasal Adenovirus expressing *M*. *tuberculosis* Ag85A increased protection against pulmonary TB infection [19]. The lack of luminal T cells in the airway following parenteral BCG may contribute to delayed protection in the lungs against *M*. *tuberculosis* [20].

The tropism of IAV to the respiratory tract [21] and the availability of licensed intranasal influenza vaccines for use in humans [22] render it a promising potential vector to improve BCG-induced immunity in the lungs. Intranasal immunization with recombinant IAV (rIAV) PR8 (H1N1) expressing the p25 CD4^+^ T cell epitope of *M*. *tuberculosis* Ag85B (PR8-p25) was immunogenic and protective against *M*. *tuberculosis* infection [23]. Sequential immunization with H1N1 (PR8-p25) and H3N2 (X31-p25) rIAV expressing the same epitope induced stronger cytokine production by p25-specific T cells and improved protective efficacy in the spleen compared to a single rIAV vaccine [24]. Importantly, pulmonary immunization with PR8-p25 and X31-p25 stimulated antigen-specific CD4^+^ lung-resident memory T cells that could confer protection against *M*. *tuberculosis* in the absence of circulating memory T cells [25]. The present study investigated the impact of pulmonary immunization with these rIAV vaccines in both SIM and prime-boost strategies with BCG on the induction of T cell responses in the lung and protection against *M*. *tuberculosis* challenge.

## Materials and methods

### Mice

Six- to eight-week old female C57BL/6 mice were purchased from the Animal Resources Centre (Perth, Australia) or Australian BioResources (Moss Vale, Australia). Mice were maintained under specific pathogen-free conditions at the Centenary Institute Animal Facility. All murine experiments were approved by Royal Prince Alfred Hospital Animal Ethics Committee (2013/075 and 2016/044).

### Recombinant influenza A viruses (rIAVs)

The IAV PR8 (H1N1, A/Puerto Rico/8/1934) and the IAV X31 (H3N2, A/HKx31) were engineered to express the *M*. *tuberculosis* p25 CD4^+^ T cell epitope (PR8-p25 and X31-p25 respectively) of Ag85B, the secreted fibronectin binding protein B encoded by *rv1886c*, as previously described [23]. In summary, the sequence encoding the 15 amino acids of the *M*. *tuberculosis* Ag85B_240-254_ CD4^+^ T cell epitope (FQDAYNAAGGHNAVF) was inserted into the gene encoding the neuraminidase (NA) stalk of the PR8 or X31 viruses and the recombinant influenza A viruses rescued using an eight-plasmid reverse genetics system [26–28]. Sequence analysis of recovered virus confirmed integrity of the insert.

### Bacteria and growth conditions

*M*. *bovis* BCG Pasteur and *M*. *tuberculosis* H37Rv strain were grown in Middlebrook 7H9 (Difco) medium supplemented with glycerol (0.2% v/v), tyloxapol (0.02% v/v) and albumin-dextrose-catalase (ADC; 10% v/v). The mycobacteria were enumerated by plating serial dilutions of organ homogenates onto 7H11 (Difco) agar supplemented with oleic-acid-albumin-dextrose-catalase (OADC; 10% v/v) and glycerol (0.5% v/v).

### Immunization and *M*. *tuberculosis* challenge

Mice were immunized by subcutaneous (s.c) injection at the base of the tail with 5x10^5^ colony forming units (CFU) BCG Pasteur in 200 μl of phosphate buffered saline (PBS) following brief isoflurane anaesthesia. For intranasal (i.n.) immunization, mice were anaesthetized by intraperitoneal (i.p.) injection with ketamine/xylazine solution (125 mg and 8 mg/Kg) and received 20 plaque forming units (PFU) PR8-p25 or 10^4^ PFU X31-p25 [23, 29] in 50 μl PBS. For SIM, parenteral BCG and i.n. PR8-p25 vaccines were delivered at the same time. For the prime-boost strategy, mice were primed with BCG 12 weeks prior to sequential immunization with rIAVs delivered at a six-week interval. Mice were challenged four weeks after the last vaccination with aerosol *M*. *tuberculosis* H37Rv with approximately 100 CFU using an inhalation exposure system (Glas-Col, Terre Haute, IN) [23]. Four weeks later, the mice were euthanized by CO2 narcosis and the bacterial loads in the lungs and spleens were enumerated. Following rIAV immunization, mice were monitored daily between 5–15 days post-immunization (d.p.i.) and otherwise twice weekly. If mice lost more than 30% of their starting body weight or met defined criteria, they were euthanized. Following *M*. *tuberculosis* infection, mice were monitored twice weekly and if they lost more than 15% of their starting body weight or met defined criteria, they were euthanized.

### Cell preparation

Prior to collecting cells for immunological assays, the lungs were perfused by injecting 10 ml PBS into the right atrium of the heart to remove blood cells from the pulmonary blood vessels prior to. Lung perfusion was not performed in experiments when all lung lobes were used for bacterial counts following *M*. *tuberculo*sis infection. Single cell suspensions were prepared from lungs and spleen. Lungs were digested by incubation with Collagenase type IV (50 U/ml, Sigma, St Louis, MO) and DNAse I (13 μg/ml, Sigma) in RPMI/10% FCS for 45 minutes at 37 °C. The lungs and spleen were disrupted by passaging the tissues through 70 μm cell strainers, washed and incubated with ACK lysis buffer to remove red blood cells. The washed cells were resuspended in RPMI/10% FCS.

### IFN-γ enzyme-linked immunospot (ELISpot) assay

IFN-γ ELISpot assay was performed as previously described [23, 30] using anti-IFN-γ antibody AN18 and biotinylated anti-IFN-γ antibody XMG1.2. The cells (2x10^5^/well) were cultured at 37 °C for 18 hours in the presence of Influenza A NP_366-374_ peptide (NP; Genscript, Piscataway, NJ), *M*. *tuberculosis* Ag85B_240-254_ peptide (p25; Genscript), *M*. *tuberculosis* H37Rv culture filtrate protein (CFP, BEI Resources), and BCG lysate at a final concentration of 10 μg/ml. The spot-forming cells (SFC) were enumerated using an automated ELISpot reader (AID, Strassberg, Germany).

### Flow cytometry analysis

Leukocyte populations and cytokine production were analysed as previously described [23, 24] using antibodies to the following cell surface markers: anti- CD3-PerCPCy5.5 (Biolegend, San Diego, CA), anti-CD4 AF700 (BD Pharmingen), anti-CD44 FITC (BD Pharmingen), anti-CD69 PE (BD Pharmingen), anti-KLRG1 PECy7 (eBioscience, San Diego, CA), and live/dead fixable staining (Life Technologies, Carlsbad, CA). For intra-cytoplasmic cytokine staining (ICS), lung and spleen cells were incubated with p25 peptide (10 μg/mL) at 37 °C for 6 hours with addition of Brefeldin A (10 μg/mL, Sigma) for the last 3.5 hours. After surface staining, the cells were washed, fixed and permeabilized with Cytofix (100 μl, BD Biosciences). The cells were then stained with anti-cytokine antibodies: anti-IFN-γ FITC (BD Pharmingen), anti-IL-2 APC (Biolegend), and anti-TNF PE (Biolegend). For analysis of cells following *M*. *tuberculosis* infection, the stained cells were re-suspended in 100 μl 4% formalin for at least 2 hours and then transferred into a new plate that was sprayed with disinfectant (3% Vircon) externally for safe transfer and acquisition on the flow cytometer outside the PC3 facility. Cells were acquired using LSR Fortessa cytometer (BD, Franklin Lakes, NJ) and analyzed using FlowJo Macintosh (Tree Star, Ashland, OR). Cytokine expression was determined using the gating strategy in S1 Fig and analyzed using the FlowJo Boolean gating tool.

### Statistical analysis

Data analyses were performed using GraphPad Prism 7 software (GraphPad Software, La Jolla, CA). Significant differences between more than two groups were analyzed by analysis of variance (ANOVA) with Tukey’s correction for multiple comparisons if the data were normally distributed or Kruskal-Wallis test with Dunn’s multiple comparison test if the data were not normally distributed based on Saphiro-Wilk test. Statistical differences with p<0.05 were considered significant.

## Results

### Antigen-specific CD4^+^ T cell response following simultaneous immunization with BCG and rIAV vaccine

**Fig 1:**
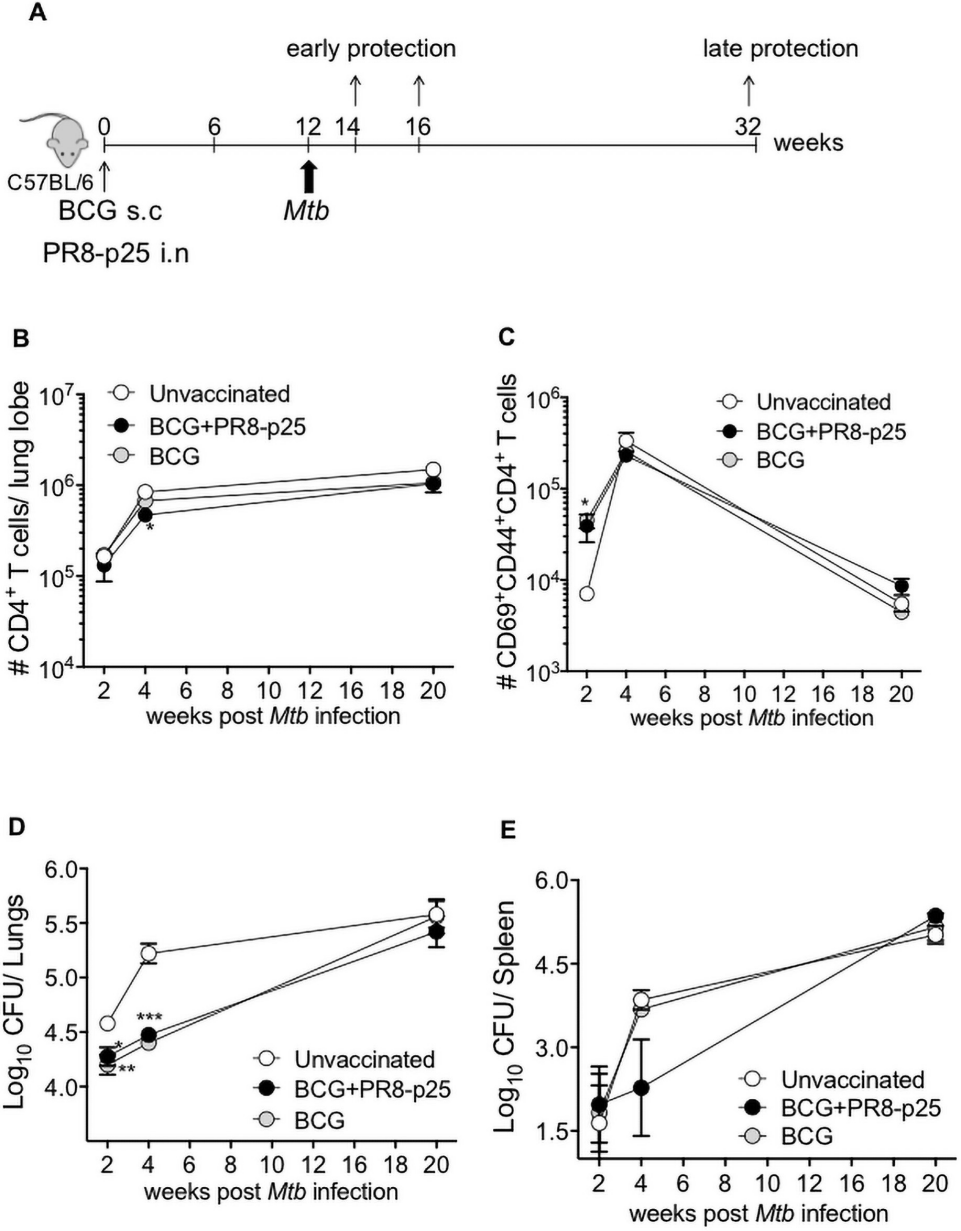
CD4^+^ T cell influx and protection against *M*. *tuberculosis* following SIM BCG and PR8-p25. (A) Experimental design for simultaneous immunization (SIM) with BCG and PR8-p25 rIAV. C57BL/6 mice (n = 5) were simultaneously vaccinated with 5x10^5^ CFU of s.c. BCG and 20 PFU of i.n. PR8-p25, s.c. BCG alone, or were left unvaccinated. The mice were challenged with *M*. *tuberculosis* 12 weeks later. Flow cytometry T cell analysis and enumeration of bacterial load were evaluated at 2, 4, and 20 weeks after *M*. *tuberculosis* challenge. (B) The kinetics of the influx of CD4^+^ T cells and (C) CD69^+^CD44^+^ CD4^+^ T cells in the lungs infected with *M*. *tuberculosis*. The kinetics of the bacterial burden are shown for the (D) lungs and (E) spleen. Data are the means ± SEM. Statistically significant differences were determined by one way-ANOVA (*p<0.05;**p<0.01; ***p<0.001).

SIM with parenteral and mucosal vaccines has been proposed as a way to improve the protective effect of immunization against *M*. *tuberculosis* in the lungs at both early and late phases of infection [14]. To investigate this strategy using BCG and rIAV, C57BL/6 mice were vaccinated with s.c. BCG and i.n. PR8-p25 simultaneously (BCG+PR8-p25) or with s.c. BCG alone. Twelve weeks later, the mice were challenged by aerosol with *M*. *tuberculosis* H37Rv, and the cellular immune responses in the lungs and protective efficacy were measured at 2, 4, and 20 weeks after challenge (Fig 1A).

Following *M*. *tuberculosis* infection, there was an influx of CD4^+^ T cells into the lungs that peaked at 4 weeks post-infection (p.i.) and was sustained for 20 weeks (Fig 1B). In both groups of vaccinated mice there were significantly increased numbers of CD4^+^ T cells expressing activation markers, CD44 and CD69, as compared to unvaccinated mice (p<0.05, Fig 1C) two weeks after *M*. *tuberculosis* infection. The frequency of activated CD4^+^ T cells peaked at four weeks and fell by 20 weeks (Fig 1C). Chronic *M*. *tuberculosis* infection is associated with terminal differentiation of activated T cells characterized by the expression of killer cell lectin-like receptor G1 (KLRG1) [31]. The kinetics of expression of KLRG1 by CD4^+^ T cells in the lungs was similar to that of the above activation markers, and there were no significant differences between the vaccinated groups (S2 Fig).

**Fig 2:**
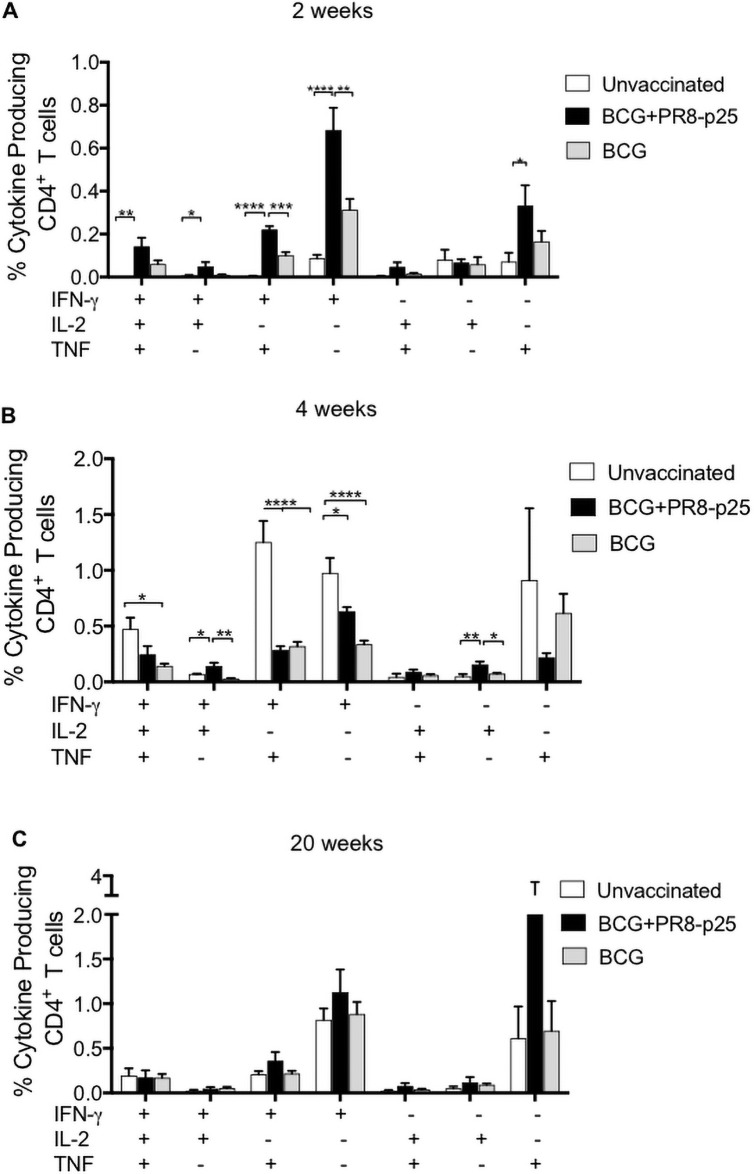
Cytokine production by p25-specific CD4^+^ T cells in the lungs after *M*. *tuberculosis* challenge. C57BL/6 mice (n = 5) were vaccinated simultaneously with s.c. BCG and i.n. PR8-p25, and challenged with *M*. *tuberculosis* as in Fig 1A. The lung cells from the same mice were stimulated with p25 antigen and then analyzed for intra-cellular cytokine production by flow cytometry. The frequency of p25-specific CD4^+^ T cells secreting IFN-γ, IL-2 and TNF at (A) 2, (B) 4, and (C) 20 weeks after challenge. Data are the means **±** SEM. Statistically significant differences between groups were determined by one-way ANOVA (*) or Kruskal-Wallis (^#^) (*p<0.05; **p<0.01; ***p<0.001; ****p<0.0001).

The Ag85B_240-254_ CD4^+^ T cell epitope (p25) encoded by PR8-p25 is present in BCG as well as *M*. *tuberculosis*. Cytokine production by p25-specific CD4^+^ T cells in the lungs was analyzed at 2, 4, and 20 weeks after *M*. *tuberculosis* challenge. At two weeks, the BCG and BCG+PR8-p25 immunized mice produced early p25-specific CD4^+^ T cell cytokine responses that were dominated by IFN-γ and TNF, but included smaller, significant increases in the frequencies of IFN-γ/TNF/IL-2 and IFN-γ/TNF secreting CD4^+^ T cells (Fig 2A). The p25-specific IFN-γ (p<0.01) response was significantly higher in the mice receiving PR8-p25 and BCG than BCG alone. A similar pattern with a lower magnitude of cytokine responses was observed in the spleen (S3A Fig). Therefore, SIM led to an early and stronger recruitment of antigen-specific T cells to the lungs following *M*. *tuberculosis* infection [32–34].

By four weeks p.i. unvaccinated mice had the highest frequency of CD4^+^ T cells secreting IFN-γ and IFN-γ/TNF compared to either vaccinated group, and higher polyfunctional CD4^+^ T cells compared to the BCG alone (Fig 2B), probably driven by the higher bacterial load in unvaccinated mice (Fig 1D). A similar pattern of cytokine-producing CD4^+^ T cells was observed in the spleens of unvaccinated mice (S3B Fig). At 20 weeks p.i., there were no differences in the pattern of cytokine production of p25-specific CD4^+^ T cells between unvaccinated and vaccinated groups (Fig 2C). IFN-γ and TNF responses were the dominant responses in each group in both the lungs (Fig 2C) and spleen (S3C Fig).

### Protective efficacy following simultaneous immunization with BCG and rIAV vaccine

The protective efficacy of SIM with BCG and i.n. PR8-p25 against *M*. *tuberculosis* aerosol infection was determined at 2, 4, and 20 weeks p.i. Vaccination with BCG or BCG+PR8-p25 resulted in a significant reduction in the lungs at two and four weeks after challenge, but this effect was lost by 20 weeks p.i. (Fig 1D). There was no difference in protective efficacy between SIM BCG+PR8-p25 and BCG at 2 or 4 weeks (Fig 1D). There was a trend towards increased protection in the spleen of mice vaccinated with BCG+PR8-p25 at 4 weeks p.i., but this was not statistically significant (Fig 1E). Therefore, simultaneous immunization with rIAV-p25 did not improve the protective efficacy of BCG against acute *M*. *tuberculosis* infection and did not prevent the loss of BCG-induced protective efficacy late in infection.

### Antigen-specific CD4^+^ T cell responses following BCG and boosting rIAV vaccines

**Fig 3:**
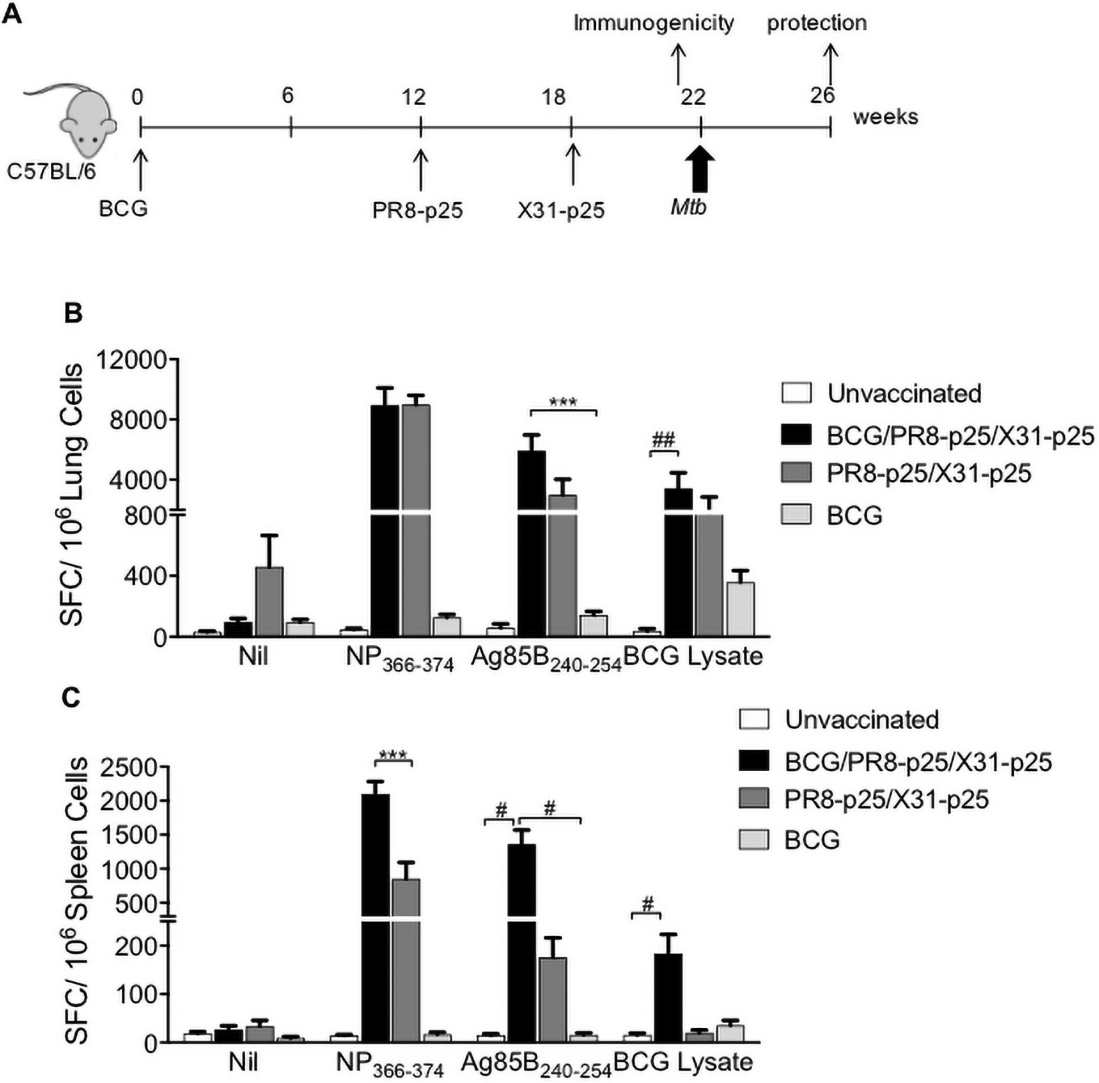
T cells producing IFN-γ in response to *M*. *tuberculosis* antigens after boosting BCG with PR8-p25 and X31-p25. (A) Experimental design for BCG boosting with PR8-p25 and X31-p25 rIAVs. C57BL/6 mice (n = 5) were vaccinated with 5x10^5^ CFU of s.c. BCG and sequentially boosted with 20 PFU of i.n. PR8-p25 and 10^4^ PFU of i.n. X31-p25. Other groups were vaccinated with BCG alone, or the two rIAVs, or were unvaccinated. (B) Four weeks after the last vaccination, the frequency of T cells in the lungs and (C) spleen producing IFN-γ in response to the CD8^+^ T cell epitope NP_366-374_ of IAV, the CD4^+^ T cell epitope Ag85B_240-254_ of *M*. *tuberculosis*, or BCG lysate, were analyzed by ELISpot as SFC/10^6^ cells. Data are the means ± SEM and are representative of two independent experiments. The differences between groups were determined by one-way ANOVA (*) or Kruskal-Wallis (^#^) (*p<0.05;**p<0.01; ***p<0.001).

To investigate if the rIAV vaccines could boost the waning protective immunity induced by BCG, BCG-vaccinated mice were sequentially immunized with HIN1 and H3N2 rIAVs expressing p25. (Fig 3A). Control mice were immunized with BCG or rIAVs alone, or were unimmunized. T cell responses in the lungs and spleen were analyzed by ELISpot and flow cytometry four weeks after the last vaccination, when the rIAVs have been cleared [35].

Boosting BCG with PR8-p25 followed by X31-p25 (BCG/PR8-p25/X31-p25) elicited a higher frequency of p25-specific T cells producing IFN-γ in the lungs, compared to vaccination with BCG alone (Fig 3B). Increased IFN-γ production was also observed following stimulation with BCG lysate that contains the Ag85B protein (Fig 3B). The response to the endogenous IAV CD8^+^ T cell epitope NP_366-374_ in the lungs of mice that received BCG prior to rIAVs was not significantly different from mice that received only the rIAVs (PR8-p25/X31-p25) (Fig 3B). Interestingly, in the spleen, increased IFN-γ T cell responses to both mycobacterial antigens and the IAV peptide antigen were seen following BCG boosting (Fig 3C). Thus, boosting BCG with intranasal rIAVs enhanced local and systemic p25-specific IFN-γ T cell responses.

**Fig 4:**
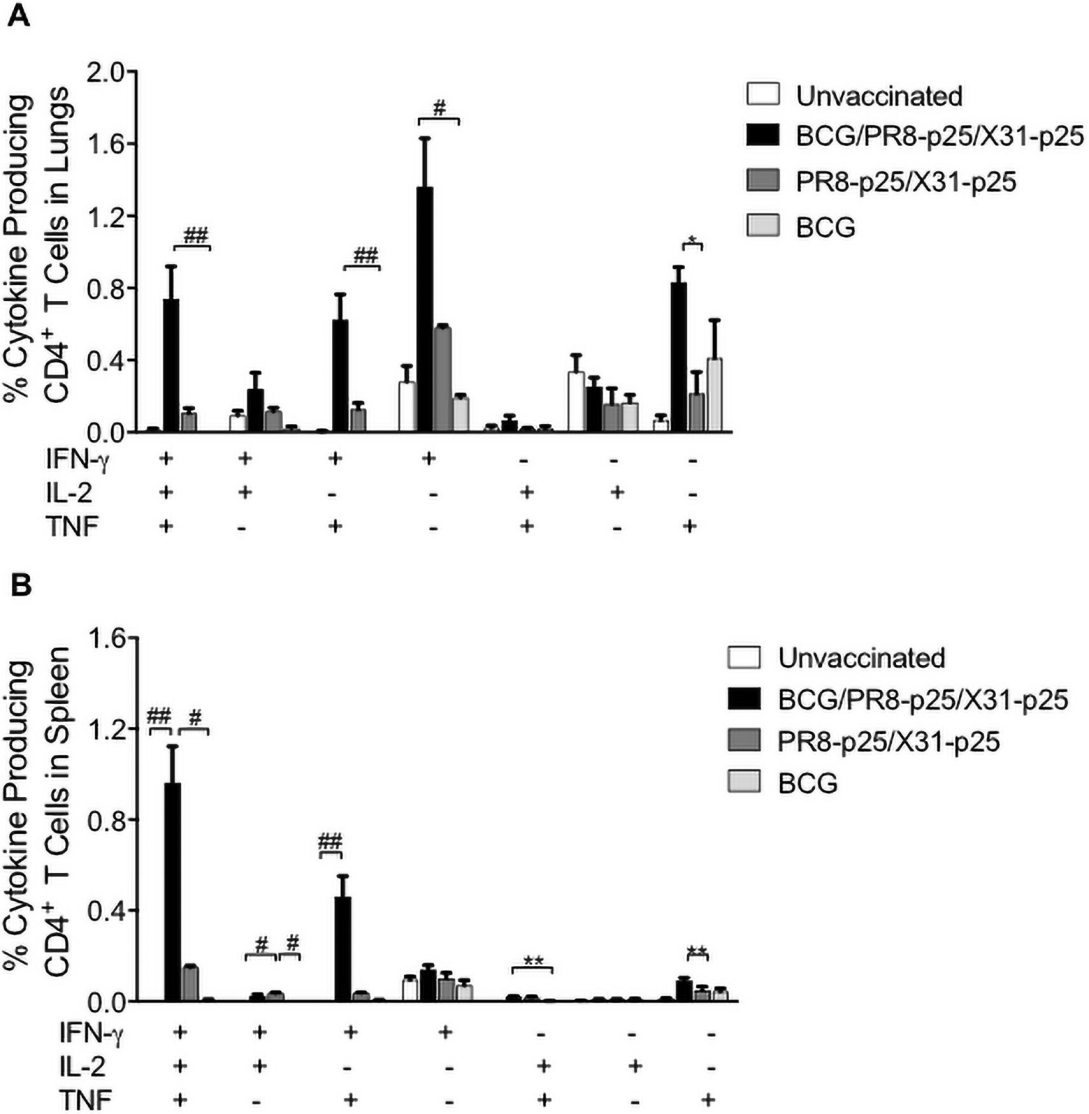
Cytokine production by CD4^+^ T cells following BCG boosting with rIAVs. C57BL/6 mice (n = 5) were vaccinated with BCG and sequential PR8-p25 and X31-p25 rIAVs as shown in Fig 3A. Four weeks after the last vaccination, lung and spleen cells were stimulated with p25 antigen and cytokine production was analyzed by flow cytometry using Boolean gating. (A) The frequency of p25-specific CD4^+^ T cells in the lungs and (B) in the spleen producing individual cytokines or combinations of IFN-γ, IL-2, and TNF. Data are the means ± SEM and are representative of two independent experiments. The differences between groups were determined by one-way ANOVA (*) or Kruskal-Wallis (^#^) (*p<0.05;**p<0.01).

BCG induced a low p25-specific cytokine response in the lungs (Fig 4A). BCG/PR8-p25/X31-p25 immunization stimulated significantly increased proportions of CD4^+^ T cell producing IFN and IFN-γ/TNF response compared to BCG (Fig 4A). Importantly, immunization with BCG/PR8-p25/X31-p25 induced the highest frequency of polyfunctional CD4^+^ T cells producing IFN-γ/IL-2/TNF in the lungs (Fig 4A). In the spleen, 1% of total CD4^+^ T cells produced triple cytokines in response to p25 stimulation, and this was significantly higher than vaccination with the BCG alone (Fig 4B). Reversing the order of the rIAV booster vaccines to BCG/X31-p25/PR8-p25 (S4A Fig) resulted in similar immunogenicity profile as BCG/PR8-p25/X31-p25. BCG/X31-p25/PR8-p25 increased p25-specific T cells secreting IFN-γ in the lungs and spleen (S4B and S4C Fig), and polyfunctional CD4^+^ T cells (S4D and S4E Fig). Therefore, boosting BCG with rIAVs stimulated stronger polyfunctional CD4^+^ T cells in the lungs and spleen than vaccination with the rIAVs or BCG alone.

### Antigen-specific CD4^+^ T cell responses following challenge with *M*. *tuberculosis*

**Fig 5:**
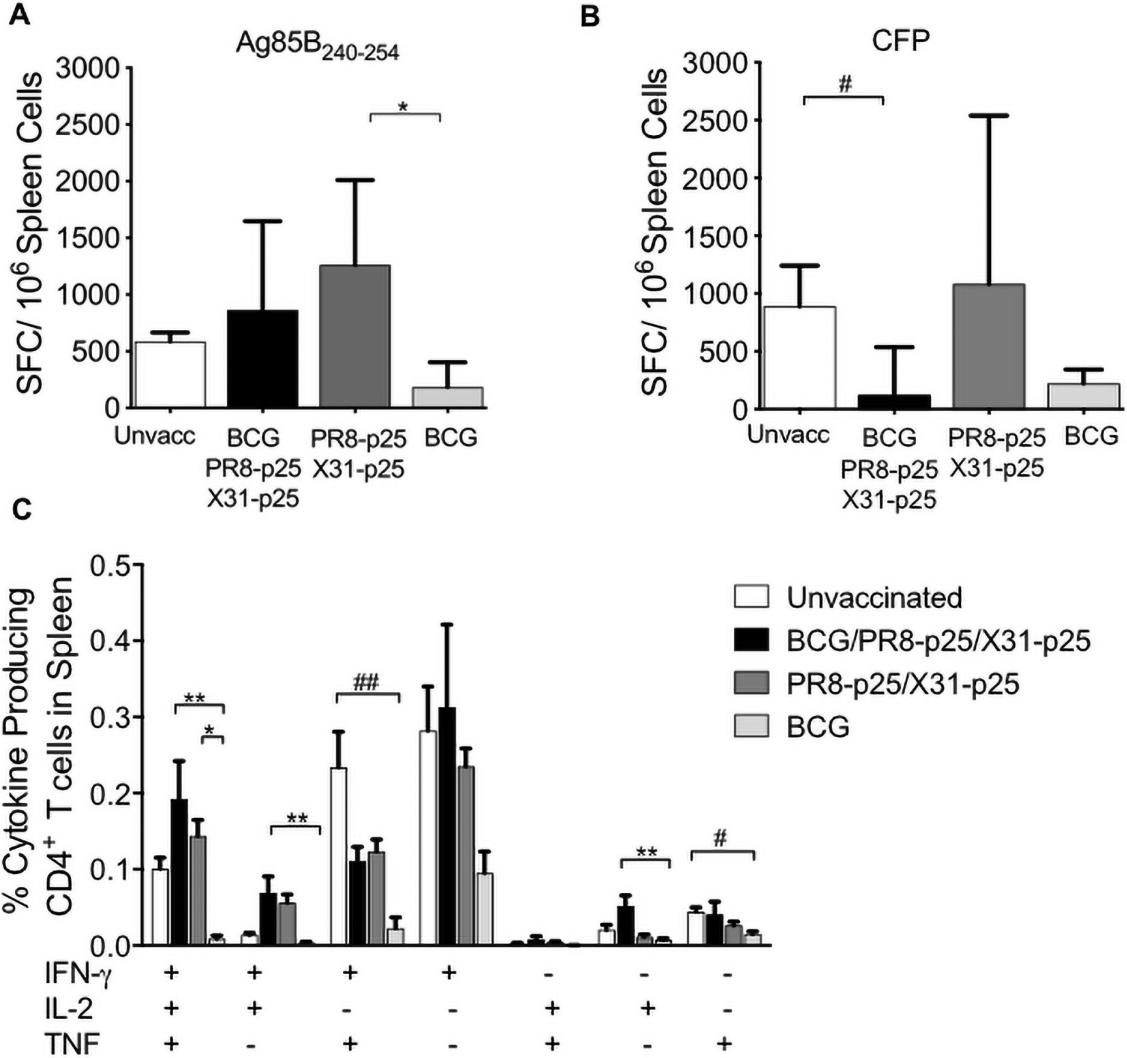
Cytokine production in the spleen at 4 weeks after aerosol challenge with *M*. *tuberculosis*. Mice were vaccinated with BCG and sequential PR8-p25 and X31-p25 rIAVs and challenged with *M*. *tuberculosis* as in Fig 3A. Four weeks after challenge, cytokine production by antigen-specific splenic CD4^+^ T cells was analyzed. (A) The frequency of T cells producing IFN-γ in response to 16-hour stimulation with p25 peptide, or (B) CFP, derived from *M*. *tuberculosis*, was analyzed by ELISpot. (C) The frequency of p25-specific CD4^+^ T cells producing IFN-γ, IL-2, and TNF following stimulation with p25 was analyzed by flow cytometry. Data are shown as the median and interquartile range (A, B) or the means ± SEM (C) and are representative of two independent experiments. Statistically significant differences were determined by one-way ANOVA (*) or Kruskal-Wallis (^#^) (*p<0.05;**p<0.01; ***p<0.001).

To investigate if boosting BCG with rIAVs influenced T cell responses following *M*. *tuberculosis* infection, cytokine production by splenocytes was analyzed four weeks after challenge. The frequency of T cells producing IFN-γ in response to stimulation with p25 antigen was similar in the unvaccinated, BCG, or BCG/PR8-p25/X31-p25 (Fig 5A). Following infection, unvaccinated mice also had a high frequency of T cells producing IFN-γ in response to stimulation with *M*. *tuberculosis* CFP (Fig 5B).

Analysis of intra-cellular cytokine expression showed unvaccinated and vaccinated mice had similar frequencies of CD4^+^ T cells producing IFN-γ alone (Fig 5C). The unvaccinated group had a higher frequency of CD4^+^ T cells producing IFN-γ/TNF compared to the BCG alone. The BCG/PR8-p25/X31-p25 group had a higher frequency of CD4^+^ T cells producing IL-2 either as an individual cytokine or in combination with IFN-γ. Interestingly, the frequency of polyfunctional CD4^+^ T cells was significantly higher in the spleens of mice receiving PR8-p25/X31-p25 or BCG/PR8-p25/X31-p25 compared to BCG alone (Fig 5C). Thus, boosting BCG with rIAVs maintained higher frequency of polyfunctional p25-specific CD4^+^ T cells than BCG alone in the spleen following four weeks *M*. *tuberculosis* infection.

### Protective efficacy following BCG and boosting rIAV vaccine

**Fig 6:**
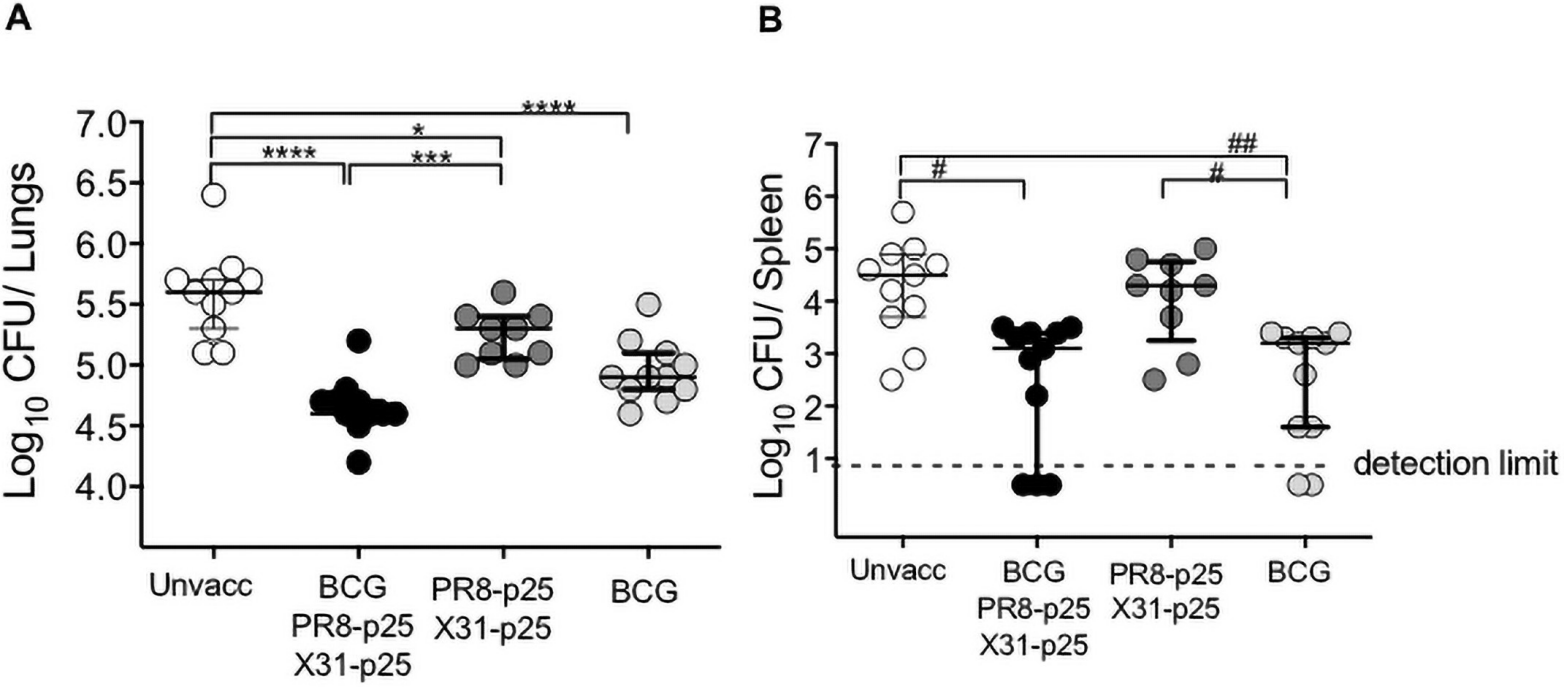
Protective efficacy of BCG boosting with PR8-p25 and X31-p25 against *M*. *tuberculosis* infection. C57BL/6 mice (n = 5–6) were vaccinated with BCG and sequentially boosted i.n. with PR8-p25 and X31-p23 as shown in Fig 3A. Four weeks after the last vaccination, mice were aerosol challenged with *M*. *tuberculosis* H37Rv, and the bacterial loads in (A) the lungs and (B) spleen were measured four weeks later. The data are pooled of two independent experiments and are shown as individual values in median and interquartile range. The differences between groups were determined by one-way ANOVA for panel A (*) or Kruskal-Wallis for panel B (^#^) (*p<0.05;**p<0.01; ***p<0.001;****p<0.0001).

To determine if boosting with rIAVs improved the protection mediated by BCG, mice were vaccinated with BCG alone or BCG followed by the rIAV vaccines (Fig 3A) were challenged with *M*. *tuberculosis* H37Rv and bacterial loads determined four weeks later. Immunization with BCG afforded protective efficacy in the lungs as compared to unvaccinated mice (p<0.0001) (Fig 6A). Mice vaccinated with rIAVs alone also had reduced bacterial counts compared to the unvaccinated group (p<0.05) (Fig 6A). Boosting BCG with the rIAVs resulted in a significant reduction of the bacterial load in the lungs compared to the unvaccinated (p<0.0001) and rIAVs (p<0.01) vaccinated groups. There was a trend towards increased protection against *M*. *tuberculosis* in the lungs of mice that received BCG boosted with rIAVs compared BCG alone in two independent experiments, but this did not reach statistical significance (Fig 6A). Mice immunized with BCG and BCG/PR8-p25/X31-p25 showed significant reductions in *M*. *tuberculosis* load in the spleen, with greater variation in the level of dissemination in the BCG-boosted mice (Fig 6B). Therefore, boosting BCG by mucosal immunization with rIAV vaccines expressing the immunodominant p25 epitope increased specific CD4^+^ T cell responses in the lung, but this was not associated with a significant increase in protection against *M*. *tuberculosis*.

## Discussion

Vaccination with BCG at birth will be retained in regions with high burden of TB because BCG protects infants from severe TB disease [4, 36]. The lack of long-term immunity following peripheral BCG vaccination, however, requires new approaches to overcome the waning of BCG-induced immunity. Developing a novel vaccine delivery mechanism, such as a viral vector administered by the pulmonary route, has been recognized as a promising approach in preclinical TB vaccine research [37]. Here, we have investigated the ability of the intranasal rIAV vaccines in SIM and prime-boost strategies to improve BCG-induced immunity.

Multiple studies show that peripheral TB vaccination induces a low frequency of T cells in the airway lumen [38]. SIM with parenteral and mucosal vaccines is proposed to induce effective protective immunity against early and late phase of *M*. *tuberculosis* infection owing to the synergistic effect of both local and systemic immune responses [14]. The current study showed that SIM with parenteral BCG and intranasal PR8-p25 did result in the early induction of higher frequency of p25-specific CD4^+^ T cells than BCG (Fig 2A), but the vaccine protection efficacy was equivalent to that provided by BCG alone (Fig 1D). This result is different to a previous finding that SIM with BCG and intranasal adjuvanted vaccine containing 85A protein, TB10.4 protein or ESAT 6 peptide, induced stronger protection *M*. *tuberculosis* than parenteral BCG [19]. Further, when intranasal BCG was combined with parenteral BCG, this provided the highest protective efficacy compared to single route of BCG [19]. The current rIAV vaccines expressed a single CD4^+^ T cell epitope, and more diverse *M*. *tuberculosis* antigens may be critical for achieving additional protection with SIM. Although SIM with BCG and PR8-p25 was able to inhibit the rapid growth of *M*. *tuberculosis* during the first four weeks of infection, this inhibitory effect waned by the chronic stage of infection, assessed at 20 weeks after challenge. Further, in cattle SIM with subcutaneous BCG and endobronchial BCG or with subcutaneous BCG and endobronchial Ad85A significantly reduced the lung pathology score, but did not decrease the bacterial load as compared to unvaccinated cattle after *M*. *bovis* challenge [39]. Thus, the potential benefit of SIM strategy compared to a single BCG vaccination will require more consistent evidence before clinical implementation.

Prime-boost immunization with BCG and the rIAVs significantly increased *M tuberculosis*-specific T cell responses compared to BCG alone or SIM with BCG/rIAV. Antigen-specific IFN-γ T cell responses in the lungs peaked at 8 weeks and contracted by 16 weeks after subcutaneous BCG immunization [9]. By contrast, intranasal boosting with PR8-p25 followed by X31-p25, or with the reverse order, prevented waning of BCG-induced immunity T cell and significantly enhanced lung and systemic p25-specific IFN-γ T cell responses for 22 weeks after the BCG. Intranasal boosting with AdHu5 expressing Ag85A also enhanced antigen-specific responses in the lung [9]. TNF production in the lung from macrophages and T cells is essential for the control of TB infection, however, the excessive TNF may contribute to immunopathology [40, 41]. The choice of viral vector may be an important factor in eliciting strong immune responses in the lungs. For instance, aerosol delivery of MVA85A vaccine failed to induce stronger Ag85A-specific responses in the lungs of BCG primed-macaques as compared to the responses following intradermal MVA85A [18]. The recombinant Influenza A virus vector utilized in this study may offer an advantage because of its tropism for the respiratory tract.

Pulmonary delivery of other non-viral TB vaccines has also been found to induce protective immunity against TB. For example, intratracheal delivery of conjugate vaccines of the Toll-like Receptor (TLR)-2 ligand, Pam2Cys, bound to either CD4^+^ and CD8^+^ T cell epitopes of *M*. *tuberculosis* [42] or to the secreted proteins ESAT-6 or MTP83 [43, 44] stimulated IL-17 and IFN-γ secreting T cell responses and protection against *M*. *tuberculosis*. Pulmonary immunization promotes development of *M*. *tuberculosis-*specific IL-17 CD4^+^ T cell responses, which in some instances are essential for the protection. For example, pulmonary, but not subcutaneous, immunization with BCG stimulated protection in the highly susceptible DBA/2 strain of mice, and this effect was IL-17 dependent [45]. Similarly, the protection conferred by pulmonary delivery of the CysVac2 protein/Advax vaccine against TB infection was also IL-17-dependent [13].

The location of memory T cells in the respiratory tract may impact the capability of T cells to respond rapidly against pulmonary *M*. *tuberculosis* infection. Early protection against *M*. *tuberculosis* infection is dependent on T cell activation and proliferation in lung-associated lymph nodes [46]. Substantial recruitment of T cells to the airway lumen could generate a suitable T cell pool for early protection [14, 38]. In addition, there is increasing evidence of the protective efficacy of tissue resident memory T cells (TRM) located in the lung parenchyma against *M*. *tuberculosis* infection following mucosal immunization with BCG [47], rIAV [25] and a protein vaccine [13] contributes to protection. However, mucosal homing of TB vaccine-induced T cells may also occur without direct mucosal immunization with some TB vaccines. For example, both mucosal and peripheral immunization with the H56:CAF01 TB vaccine that contains the adjuvant, trehalose dibenalate, stimulated vaccine-specific Th1 and Th17 T cells that homed to the lung [48]. Recently, intravenous delivery of BCG in the non-human primate, *Macaca mulatta*, was demonstrated to stimulate the highest numbers of BCG-specific CD4^+^ and CD8^+^ memory T cells in the bronchoalveolar lavage (BAL) and lung lymph nodes and significantly enhanced protection against *M*. *tuberculosis* compared to aerosol or intradermal immunization with BCG [49]. Therefore, comprehensive investigation of the immune response in the lung compartments, BAL, and lung-associated lymph nodes is essential to elucidate the mechanism of protective immunity induced by mucosal or non-mucosal immunization with individual TB vaccines and when combined with BCG in prime-boost regimens.

Protective biomarkers for TB vaccines have proved difficult to define (reviewed in [50]). Prime-boost vaccination with BCG and rIAVs generated a high frequency of CD4^+^ T cells producing IFN-γ, IL-2, and TNF in the lungs and spleen. An increase in polyfunctional CD4^+^ T cells following BCG boosting with viral TB vaccines has been previously correlated with protection against *M*. *tuberculosis* with some [17, 51], but not all, TB vaccines [50]. For example, the increased level of polyfunctional CD4^+^ T cells following BCG boosting with the Nano-FP1 mucosal vaccine correlated with an early but not long-term increase in protective efficacy compared to BCG alone [52]. Although the mechanism is unclear, polyfunctional cells are proposed to be long-lived cells with potent anti-bacterial activity [53, 54]. In the current study, the significant increase in the frequency of p25-specific T cell producing multiple cytokines in the lungs generated by boosting BCG with rIAVs did not correlate with a significant increase in the protection of lungs from TB compared to the BCG alone. Improving the magnitude and the quality of T cell responses that are specific to a single antigen by mucosal boosting may not be sufficient to stimulate significant additional protection to BCG. The responses to BCG vaccine are complex and it is possible that BCG could induce “decoy immune” responses to dominant BCG antigens that prevent the induction of preventive immune response to boosting vaccines [55]. Therefore, the selection of antigens for boosting vaccines is important, and could include *M*. *tuberculosis* proteins that are either missing from BCG or are non-dominant antigens in BCG. For example, immunization with the M72 fusion protein vaccine containing the *M*. *tuberculosis* 32A and 39A antigens was found to provide 50% protection against developing active pulmonary TB in adults with latent TB infection [56]. The inclusion of additional *M*. *tuberculosis* antigens in the rIAV vaccines can be developed by manipulation of HA, NA and NS segment of rIAV to carry other *M*. *tuberculosis* antigens [23, 57, 58]. As a feasible model for an intranasal vaccine, further investigation on the cellular immune responses developed in the lungs following immunization with rIAV expressing multiple antigens may provide insight into the advantages of pulmonary TB vaccine.

## Conclusion

This study demonstrates that mucosal vaccination with rIAV expressing single *M*. *tuberculosis* epitope could increase cytokine production by antigen-specific T cells following co-delivery or priming with parenteral BCG. Prime-boost immunization has greater potential as a strategy to improve BCG-induced protective immunity than simultaneous immunization. The inclusion of additional *M*. *tuberculosis* antigens in the rIAV may result in significant improvement on boosting the protective efficacy of BCG.

## Supporting information

S1 Fig Gating strategy for flow cytometry analysis. Single events were gated using forward scatter (FSC-A/FSC-H) and side scatter (SSC-A/SSC-H) flow plots. Dead cells were excluded and viable cells were gated for the lymphocyte population. The CD4^+^ T cell population was gated as CD3^+^CD4^+^ lymphocytes. For surface staining, T cell and differentiation activation markers were gated as CD44^+^CD69^+^ or CD4^+^KLRG1^+^ within the CD4^+^ T cell population. For ICS, the antigen-specific CD4^+^ T cells secreting IFN-γ, IL-2, or TNF were identified and analyzed using the FlowJo Bolean gating tool.
(TIFF)

S2 Fig KLRG1 CD4^+^ T cell following SIM BCG and PR8-p25. C57BL/6 mice (n = 5) were simultaneously vaccinated with 5x10^5^ CFU of s.c. BCG and 20 PFU of i.n. PR8-p25, s.c. BCG alone, or were left unvaccinated. The mice were challenged with *M*. *tuberculosis* 12 weeks later. KLRG1+CD44+CD4+ T cells in the lungs at two, four, and twenty weeks after *M*. *tuberculosis* challenge. Data are the means ± SEM. Statistically significant differences were determined by one way-ANOVA (p<0.05).
(TIFF)

S3 Fig Cytokine production by p25-specific CD4^+^ T cells in the spleen after *M*. *tuberculosis* challenge. C57BL/6 mice (n = 5) were vaccinated simultaneously with BCG s.c. and PR8-p25 i.n., and challenged with *M*. *tuberculosis* as in Fig 1A. The splenocytes were stimulated with p25 antigen and then analysed for intra-cellular cytokine production by flow cytometry. The frequency of p25-specific CD4^+^ T cells secreting IFN-γ, IL-2 and TNF were detrermined at (A) two, (B) four, and (C) twenty weeks after challenge. Data are the means **±** SEM. Statistically significant differences between groups were determined by one-way ANOVA (*) or Kruskal-Wallis (^#^) (*p<0.05; **p<0.01).
(TIFF)

S4 Fig Antigen-specific T cell responses following BCG boosting with X31-p25 and PR8-p25. (A) Experimental design for BCG boosting with X31-p25 and PR8-p25 rIAVs. C57BL/6 mice (n = 4–6) were vaccinated with 5x10^5^ CFU BCG s.c., vaccinated 12 weeks later with 10^4^ PFU X31-p25 i.n. and then were boosted a second time at 18 weeks with 20 PFU PR8-p25 i.n. Other groups were vaccinated with BCG alone, the two rIAVs alone, or were left unvaccinated and antigen-specific T cell responses were assessed four weeks after the last vaccination. ELISpot analysis of IFN-γ producing T cells in the (B) lungs and (C) spleen following 18 hour stimulation with the relevant antigens. (A) The frequency of p25-specific CD4^+^ T cells in the (D) lungs and (E) spleen producing individual cytokines or combinations of IFN-γ, IL-2, and TNF were analysed by ICS flow cytometry using Boolean gating. Data are the means ± SEM. Statistically significant differences between groups were determined by one-way ANOVA (*) or Kruskal-Wallis (^#^) (*p<0.05;**p<0.01; ***p<0.001; ****p<0.0001).
(TIFF)
