# Supplementary figures and images for: Boosting BCG with recombinant influenza A virus tuberculosis vaccines increases pulmonary T cell responses but not protection against Mycobacterium tuberculosis infection
Source: PLoS One. 2021 Nov 18;16(11):e0259829. doi: 10.1371/journal.pone.0259829 (PMC8601556; doi:10.1371/journal.pone.0259829)

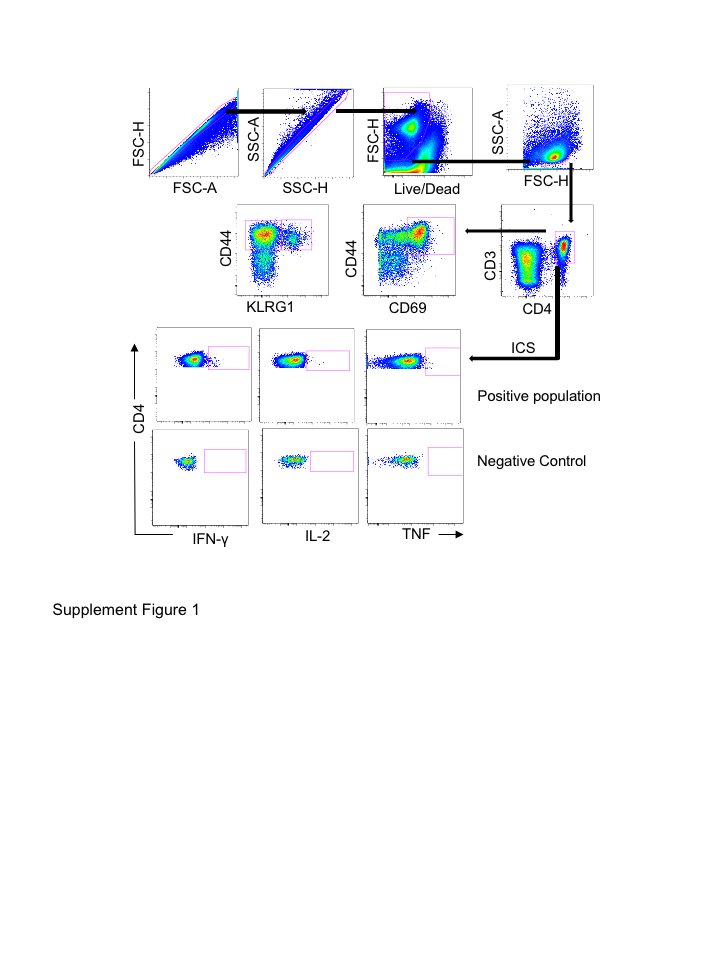

Supplement: S1 Fig — Single events were gated using forward scatter (FSC-A/FSC-H) and side scatter (SSC-A/SSC-H) flow plots. Dead cells were excluded and viable cells were gated for the lymphocyte population. The CD4+ T cell population was gated as CD3+CD4+ lymphocytes. For surface staining, T cell and differentiation activation markers were gated as CD44+CD69+ or CD4+KLRG1+ within the CD4+ T cell population. For ICS, the antigen-specific CD4+ T cells secreting IFN-γ, IL-2, or TNF were identified and analyzed using the FlowJo Bolean gating tool. (TIFF) [file pone.0259829.s001.tiff]

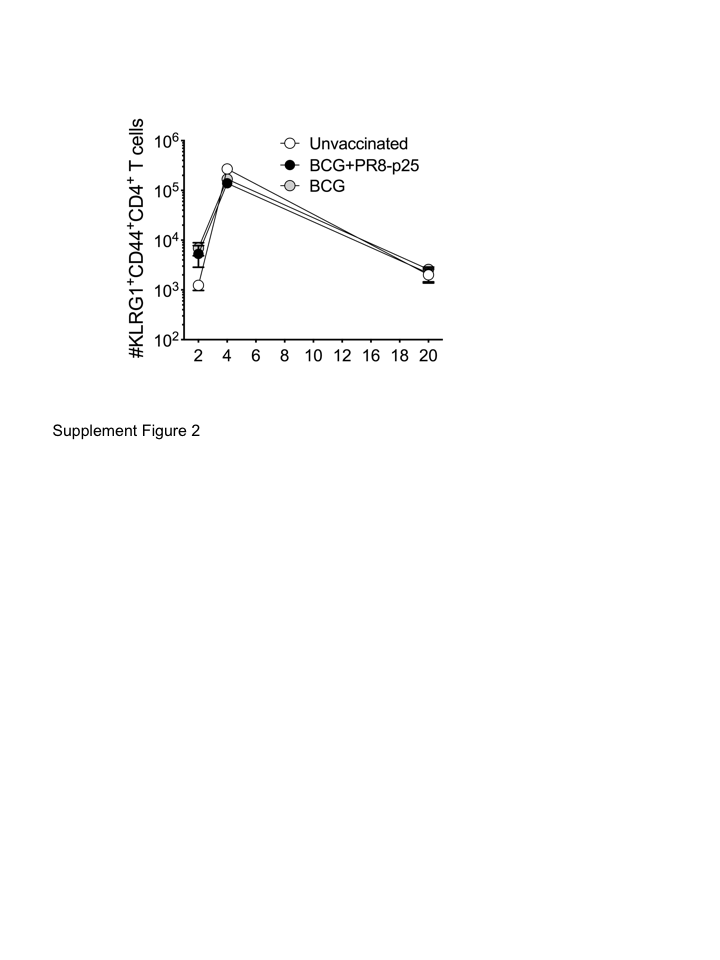

Supplement: S2 Fig — C57BL/6 mice (n = 5) were simultaneously vaccinated with 5x10^5^ CFU of s.c. BCG and 20 PFU of i.n. PR8-p25, s.c. BCG alone, or were left unvaccinated. The mice were challenged with M. tuberculosis 12 weeks later. KLRG1+CD44+CD4+ T cells in the lungs at two, four, and twenty weeks after M. tuberculosis challenge. Data are the means ± SEM. Statistically significant differences were determined by one way-ANOVA (p<0.05). (TIFF) [file pone.0259829.s002.tiff]

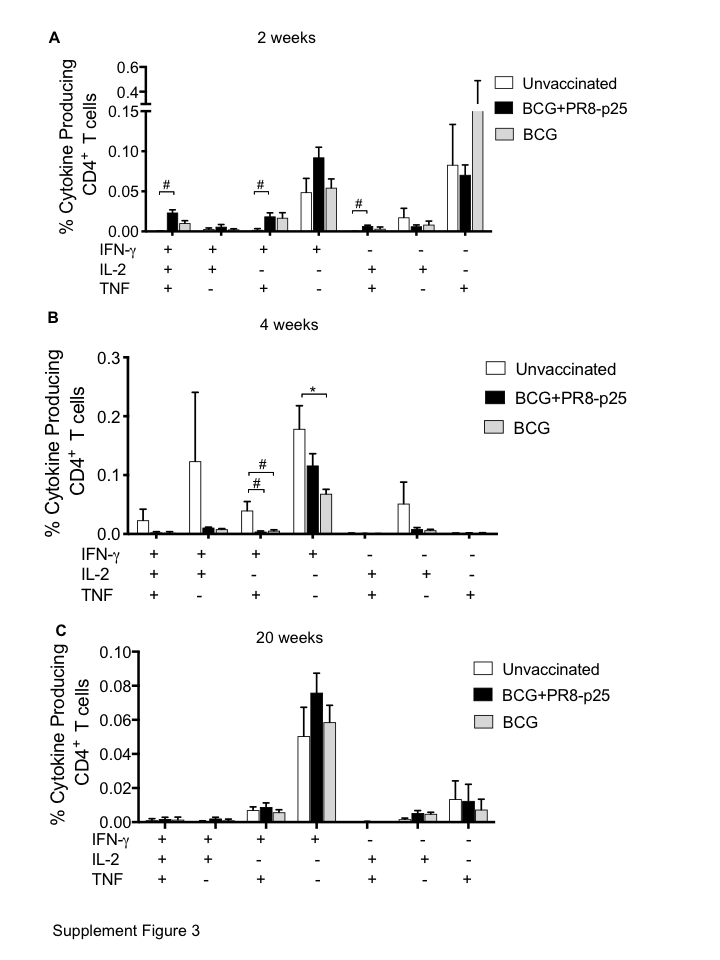

Supplement: S3 Fig — C57BL/6 mice (n = 5) were vaccinated simultaneously with BCG s.c. and PR8-p25 i.n., and challenged with M. tuberculosis as in Fig 1A. The splenocytes were stimulated with p25 antigen and then analysed for intra-cellular cytokine production by flow cytometry. The frequency of p25-specific CD4+ T cells secreting IFN-γ, IL-2 and TNF were detrermined at (A) two, (B) four, and (C) twenty weeks after challenge. Data are the means ± SEM. Statistically significant differences between groups were determined by one-way ANOVA (*) or Kruskal-Wallis (#) (*p<0.05; **p<0.01). (TIFF) [file pone.0259829.s003.tiff]

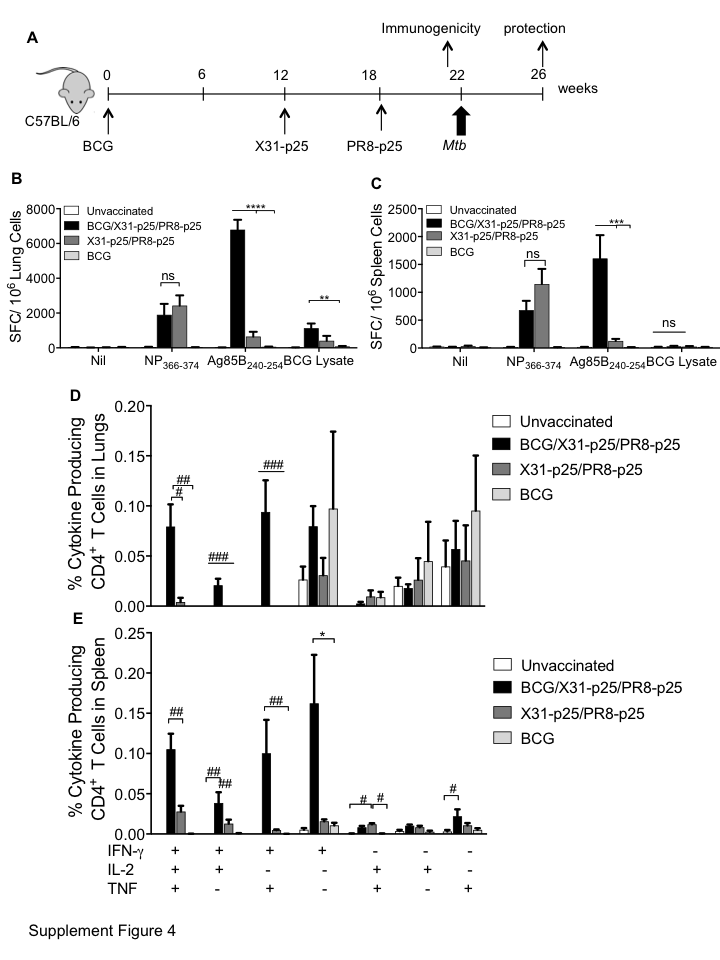

Supplement: S4 Fig — (A) Experimental design for BCG boosting with X31-p25 and PR8-p25 rIAVs. C57BL/6 mice (n = 4–6) were vaccinated with 5x10^5^ CFU BCG s.c., vaccinated 12 weeks later with 104 PFU X31-p25 i.n. and then were boosted a second time at 18 weeks with 20 PFU PR8-p25 i.n. Other groups were vaccinated with BCG alone, the two rIAVs alone, or were left unvaccinated and antigen-specific T cell responses were assessed four weeks after the last vaccination. ELISpot analysis of IFN-γ producing T cells in the (B) lungs and (C) spleen following 18 hour stimulation with the relevant antigens. (A) The frequency of p25-specific CD4+ T cells in the (D) lungs and (E) spleen producing individual cytokines or combinations of IFN-γ, IL-2, and TNF were analysed by ICS flow cytometry using Boolean gating. Data are the means ± SEM. Statistically significant differences between groups were determined by one-way ANOVA (*) or Kruskal-Wallis (#) (*p<0.05;**p<0.01; ***p<0.001; ****p<0.0001). (TIFF) [file pone.0259829.s004.tiff]
